# CX3CL1 and CX3CR1 could be a relevant molecular axis in the pathophysiology of idiopathic pulmonary fibrosis

**DOI:** 10.7150/ijms.43748

**Published:** 2020-08-29

**Authors:** Selma Rivas-Fuentes, Iliana Herrera, Alfonso Salgado-Aguayo, Ivette Buendía-Roldán, Carina Becerril, José Cisneros

**Affiliations:** 1 Department of Research on Biochemistry, Instituto Nacional de Enfermedades Respiratorias Ismael Cosío Villegas, Mexico City, Mexico.; 2 Laboratory of Cell Biology, Department of Research on Pulmonary Fibrosis. Instituto Nacional de Enfermedades Respiratorias Ismael Cosío Villegas, Mexico City, Mexico.; 3 Laboratory of Research on Rheumatic Diseases, Instituto Nacional de Enfermedades Respiratorias Ismael Cosío Villegas, Mexico City, Mexico.; 4 Laboratory of Translational Research on Aging and Fibrosis Lung, Instituto Nacional de Enfermedades Respiratorias Ismael Cosío Villegas, Mexico City, Mexico.

**Keywords:** idiopathic pulmonary fibrosis, collagen production, fibroblasts

## Abstract

Idiopathic pulmonary fibrosis is a chronic and progressive disease of unknown cause. It is characterized by the aberrant activation of the bronchioalveolar epithelium, the formation of fibroblast foci and the excessive production extracellular matrix. The cellular and molecular mechanisms that contribute to the pathobiology of the disease are unclear. The CX3CL1-CX3CR1 axis regulates cellular responses that are known to be relevant in IPF, such as proliferation and collagen production. In this study, we characterize for the first time the expression of CX3CL1 and its receptor in lung tissue from patients with IPF; and its effect on collagen production in IPF fibroblasts. We found that CX3CL1-CX3CR1 axis has a modified expression in the lung tissue, importantly this axis is expressed on fibroblasts, and CX3CL1 decreased the collagen production in pulmonary fibroblasts derived from IPF patients.

## Introduction

Idiopathic pulmonary fibrosis (IPF) is a chronic disease of unknown cause, poor prognosis and no effective therapy 1, 2. The disease is characterized by an aberrant activation of bronchioalveolar epithelium and mesenchymal cells, leading to the formation of fibroblast and myofibroblast foci, excessive production of extracellular matrix, and the consequent destruction of lung architecture 3, 4. However, the cellular and molecular mechanisms that contribute to the pathobiology of the disease are still unclear. Previous work indicates that chemokines play a role in the development of pulmonary fibrosis. For example, CCL3 bound to CCR5 is important in the recruitment of TGF-β-producing macrophages and fibrocytes into the lung in response to bleomycin 5. Using the same model, Tokuda *et al.* reported that bleomycin challenge resulted in an accumulation of CCR1^+^ cells in the interstitial inflammatory site. The inhibition of CCR1 signaling with antibodies led to a reduced accumulation of these cells, and a diminished collagen deposition 6. In humans, recent studies underscore the importance of chemokines in the progression of IPF. For example, IPF mesenchymal progenitor cells produce higher IL-8 (CXCL8) levels than controls, and this chemokine can both stimulate macrophage migration and promote the self-renewal of these progenitor cells 7.

CX3CL1 (or Fractalkine) is a chemokine that binds to the CX3CR1 receptor. This chemokine can be expressed as a membranal protein with an intracytoplasmic domain, but a soluble form can be produced by enzymatic proteolysis 8. It has been reported that stimulation with TGF-β, a critical mediator in IPF, increases the levels of CX3CR1 mRNA in primary cultures of rat microglia, suggesting that CX3CR1 could be increased in microenvironments enriched in TGF-β 9. Recent studies indicate that the CX3CL1-CX3CR1 axis is involved in several fibrotic disorders. For example, studies in animal models indicate that the CX3CL1-CX3CR1 axis directly affects the production of collagen in various types of fibrotic processes such as systemic sclerosis 10, obstructive nephropathy 11 and liver fibrosis 12.

**Figure 1:**
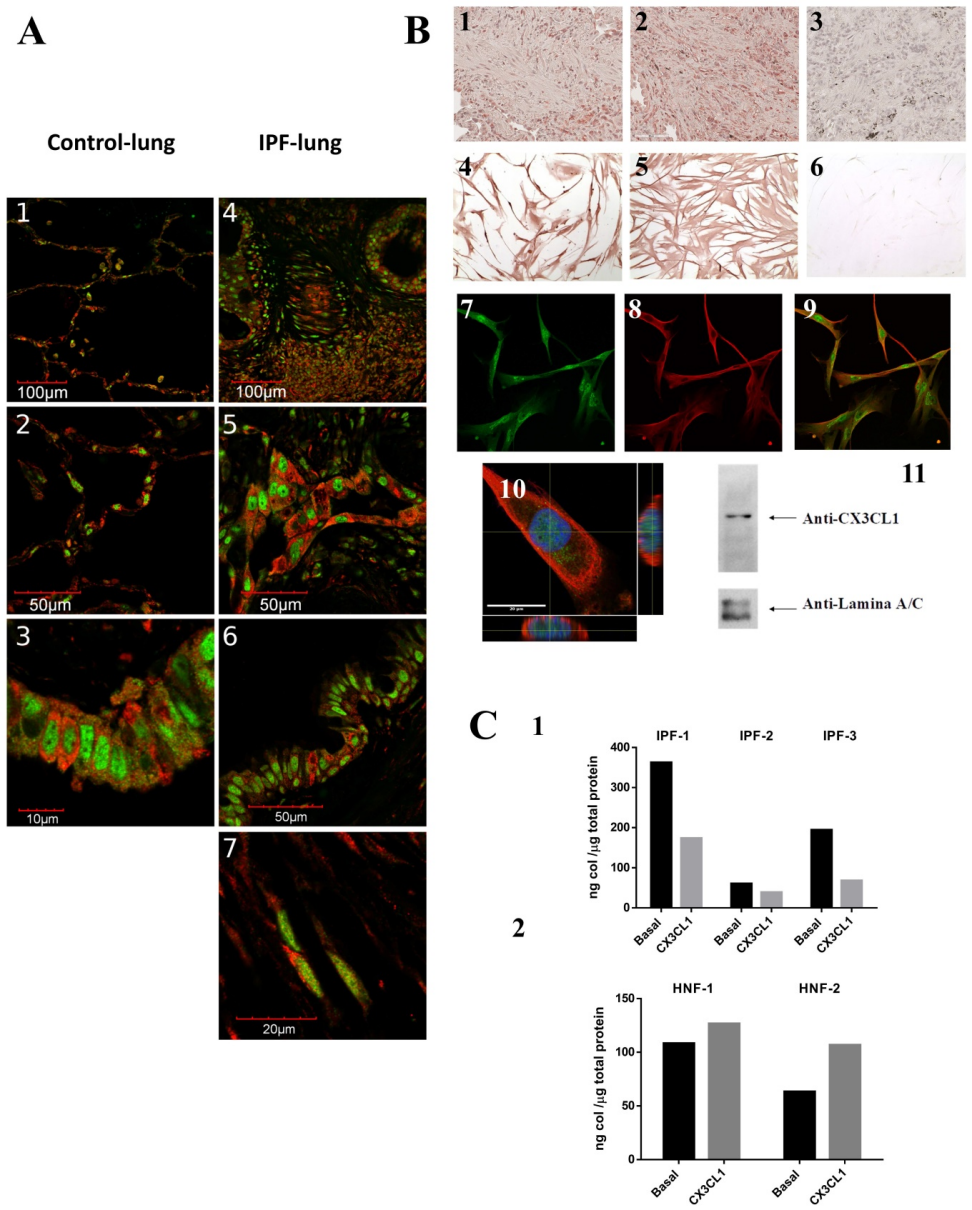
** CX3CL1 and CX3CR1 could form a relevant molecular axis in the pathophysiology of idiopathic pulmonary fibrosis. A.** The CX3CL1-CX3CR1 axis is expressed in altered alveolar epithelium and fibrotic tissue and are co-expressed on stromal cells and a subpopulation of epithelial cells. Tissues from 6 IPF and 5 non-fibrotic lungs were processed for immunohistochemistry or double immunofluorescence staining using CX3CR1 and CX3CL1 primary antibodies. Panels A1-3 shows images from a control lung: alveolar epithelium lung (A1, 2) and bronchial epithelium (A3). Panels A4-7 show images from IPF lung: alveolar epithelium and fibrotic tissue (A4), hyperplastic type II pneumocytes (A5), bronchial epithelium (A6) and fibroblast (A7). Images are representative from four different patients and two controls. **B.** CX3CL1 and CX3CR1 are expressed in fibroblast foci and isolated fibroblasts, and CX3CL1 localizes to plasma membrane and nucleus. CX3CL1 and CX3CR1 are expressed in fibroblastic foci in IPF lungs (B1 and B2, respectively). CX3CL1 is expressed in cultured IPF lung fibroblast in a membranal and nuclear localization (B4) while CX3CR1 was exclusively found in the plasma membrane (B5). Samples incubated only with secondary antibody didn´t develop staining (B3, 6). Figure is representative of five fibroblast lines. CX3CL1 and CX3CR1 expression was also evaluated by immunofluorescence in IPF fibroblast lines. Panels B7 and B8 respectively show CX3CL1 (green) and CX3CR1 (red) expression, while Panel B9 corresponds to the merged images. A putative nuclear localization of CX3CL1 is shown with a Z Stack analysis of a fibroblast stained with anti-CX3CL1 and anti-CX3CR1 antibodies, and DAPI; orthogonal views show intranuclear localization of CX3CL1 (B10); while in a western blot of nuclear extracts from IPF lung fibroblasts using an anti-CX3CL1 antibody, a band of 150 KDa was detected (B11). **C.** CX3CL1 decreases pro-collagen production from IPF derived fibroblasts but not in control fibroblast lines. C1 shows a decrease on collagen concentration in conditioned media from 3 IPF cultured lung fibroblasts stimulated by CX3CL1(100 ng/ml) during 48 hrs compared to the unstimulated counterpart. Differences between basal and CX3CL1-stimulated collagen levels were tested with Student´s ratio T-test (p <0.05). C2 shows that, regardless of the stimulus, collagen levels did not significantly change on non-fibrotic fibroblast cell line (2 lines).

In this study, we characterized for the first time in lung tissue from patients with IPF, the expression of CX3CL1 and its receptor CX3CR1, and their effects on collagen production. We analyzed by immunohistochemistry and immunofluorescence 6 samples from IPF lungs and 5 from control lungs using specific antibodies against CX3CL1 and CX3CR1 (see methods). We found that CX3CL1 and CX3CR1 are widely distributed in IPF and control lungs. On normal lung, CX3CL1 and CX3CR1 are found in the alveolar and bronchial epithelium, as well as in vascular endothelium, while on IPF lung there was also positive staining in the fibrotic tissue, in fibroblasts and in alveolar hyperplastic cells and glandular alterations (**Figure 1A**). In the fibrotic tissue, some cells expressing the receptor (shown in red) are in close proximity to cells expressing the chemokine (in green), suggesting an *in situ* CX3CR1-CX3CL1 interaction among several cell types*.* Interestingly, we found some cells co-expressing CX3CL1-CX3CR1 in control lungs as well as in hyperplastic epithelium and fibroblasts in IPF lungs, suggesting that an epithelium-fibroblast crosstalk mediated by the CX3CL1-CX3CR1 axis could be possible.

We analyzed by immunocytochemistry the CX3CL1 and CX3CR1 expression in isolated fibroblasts derived from IPF lungs and control fibroblasts. We observed a membrane and nuclear localization of CX3CL1 on normal and IPF fibroblasts **Figure 1B**_4_. Similar results were obtained by immunofluorescence as shown in **Figure 1B**_7_. Very few studies have reported a nuclear localization of chemokines, i.e. CXCL12 (SDF-1) 13, 14. We confirmed the nuclear localization of CX3CL1 with orthogonal projections of confocal microscope-obtained z-stacks **Figure 1B**_10_, as well as Western blot from the isolated nuclear fraction from IPF lung fibroblasts, assayed with an anti-CX3CL1 antibody (**Figure 1B**_11_**)**.

Studies in animal models of fibrosis indicate that the CX3CL1-CX3CR1 axis has a direct impact on the production of collagen 12, 15. Therefore, we analyzed the production of collagen in IPF lung fibroblasts and in control cell lines using the Sircol assay. Fibroblasts were stimulated for 48 hours with soluble CX3CL1. We found that IPF fibroblasts decreased their collagen production (35-63%, *p*<0.05) in the presence of CX3CL1 compared to unstimulated fibroblasts (**Figure 1C**_1_). Importantly, the production of collagen was not significantly affected in control fibroblasts (**Figure 1C**_2_). This result suggests that, similar to what is reported on liver damage, the interaction between CX3CR1 and CX3CL1 has a protective function in relation to the production of collagen 12. In contrast, in the murine model of bleomycin-induced pulmonary fibrosis, an up-regulation of the CX3CL1-CX3CR1 axis was associated with intrapulmonary accumulation of pro-fibrotic M2 macrophages, while CX3CR1^-/-^ mice exhibited a reduction of M2 macrophage recruitment and collagen production 16, suggesting a pro-fibrotic role of the CX3CL1-CX3CR1 axis. The bleomycin model has been very valuable in understanding various aspects of the pathophysiology of idiopathic pulmonary fibrosis. However, there are important differences with human IPF, the most notable being that the experimental model is self-contained and partially reversible 17, 18, which could partially explain these differences.

The presence of CX3CL1 decreased *in vitro* collagen production in IPF-derived fibroblasts but not in healthy fibroblasts. MMP-2 plays an important role in IPF physiopathology and its activity is increased on BAL from IPF patients. It is expressed by alveolar epithelial cells and fibroblasts 19. In activated human hepatic stellate cells, membrane-bound CX3CL1 is shedded by MMP-2 20. Hence, the increase of MMP-2 in cultured IPF fibroblasts could have an effect in the relative amounts of membrane-bound and soluble CX3CL1 and consequently in the functional responses to this chemokine, since both forms of CX3CL1 produce different responses 21-23. On the other hand, in primary cultures of rat microglia, TGF-β stimulation results in an increase of CX3CR1 levels. TGF-β plays a central role in the physiopathology of IPF and is increased in the lung of IPF patients. It is interesting to hypothesize that increased TGF-β levels lead to an overexpression of CX3CR1 in IPF fibroblasts, with different functional responses to CX3CL1 9.

In conclusion, we found that CX3CL1 and CX3CR1 are ubiquitously distributed in IPF lungs, including fibroblastic foci; and CX3CL1 decreased the collagen production in isolated IPF fibroblast, suggesting an anti-fibrotic role.

## Methods

### Immunofluorescence

We evaluated 4 IPF cases and 5 controls using 3 µm thick FFPE lung sections. We used rabbit anti-CX3CL1 and anti-CX3CR1, and goat anti-CX3CL1 (Abcam, Cambridge, United Kingdom) primary antibodies, detected with anti-rabbit IgG Alexa fluor (AF) 647 and anti-goat IgG AF 546 (Jackson Immunoresearch, Baltimore, USA). We analyzed 5 lung fibroblast lines derived from IPF lungs, (obtained as described in Becerril et al. 1999 24); healthy lung fibroblasts (HPF and NHLF) were obtained from Promocell (Heidelberg, Germany), Lonza (Basel, Switzerland) and primary cultures from cadaveric donation (FN-2-13). 20,000 fibroblasts were seeded in coverslips. The next day, cells were fixed with cold methanol/acetone. After blocking (1X Powerblock™ (Biogenex, Cal. USA) + 0.5% Triton X-100), samples were incubated ON with primary antibodies, then for 1 hour with fluorescent secondary antibodies, and counterstained with DAPI. Fluorescence was evaluated with an FV-1000 laser scanning confocal microscope (Olympus, Tokyo, Japan).

### Immunocytochemistry

5,000-7,000 lung fibroblast cells from individuals with IPF or lines of non-fibrotic pulmonary fibroblasts were seeded by condition in 96-well plates. Cells were stained using the aforementioned primary antibodies, which were then detected with the Mouse/Rabbit HRP DAKO biotinylated link (Agilent, Cal, USA), AEC detection and colour development system (BioGenex).

### Immunohistochemistry

FFPE samples were rehydrated, subjected to antigen retrieval with citrate buffer, and incubated in a hydrogen peroxide solution. Samples were then processed in a manner similar to that described earlier.

### WB

Nuclear extracts from IPF fibroblast were obtained with the NE-PER kit from Pierce (Thermo Fisher Scientific, Waltham, MA, USA), and CX3CL1 detected by western blot with an anti-CX3CL1 antibody (Abcam).

### Collagen determination

We determined the acid and pepsin soluble collagen on fibroblast supernatants using the Sircol assay (Biocolor Antrim, UK). 1-1.5 × 10^6^ cells seeded on 75 cm^2^ flask were used by condition. Cells were stimulated for 48 hrs with 100 ng/ml of CX3CL1 (Biolegend San Diego, USA); non-stimulated cells were also tested. After stimulation, culture medium was collected, lyophilized and reconstituted in 70 µl deionized water. The concentration of collagen in 50 µl of each sample was evaluated by the Sircol assay according to the manufacturer's instructions; each sample was run in duplicates. Samples were read at OD_550_ nm and at OD_600_ nm in an Epoch plate spectrophotometer (Biotek, Winooski, USA). Concentration of collagen was calculated with a standard curve using the Gen 5 software (Biotek).

### Statistics

Continuous variables from the immunoassay are expressed as medians; the minimum and maximum value (min, max value) are also shown. In the Sircol test the continuous variables are expressed as means ± standard error. Statistical contrasts were performed with the GraphPad Prism v6.0 software. Data from the Sircol assay were compared with Student´s ratio *t*-test. Significant differences were considered at values ​​of *p* ≤ 0.05.

## Supplementary Material

Supplementary figure S1.
