# Supplementary material for: CX3CL1 and CX3CR1 could be a relevant molecular axis in the pathophysiology of idiopathic pulmonary fibrosis
Source: Int J Med Sci. 2020 Aug 29;17(15):2357–61. doi: 10.7150/ijms.43748 (PMC7484633; doi:10.7150/ijms.43748)

Supplementary Figure: CX3CL1 and CX3CR1 could be a relevant molecular axis in the pathophysiology of idiopathic pulmonary fibrosis.

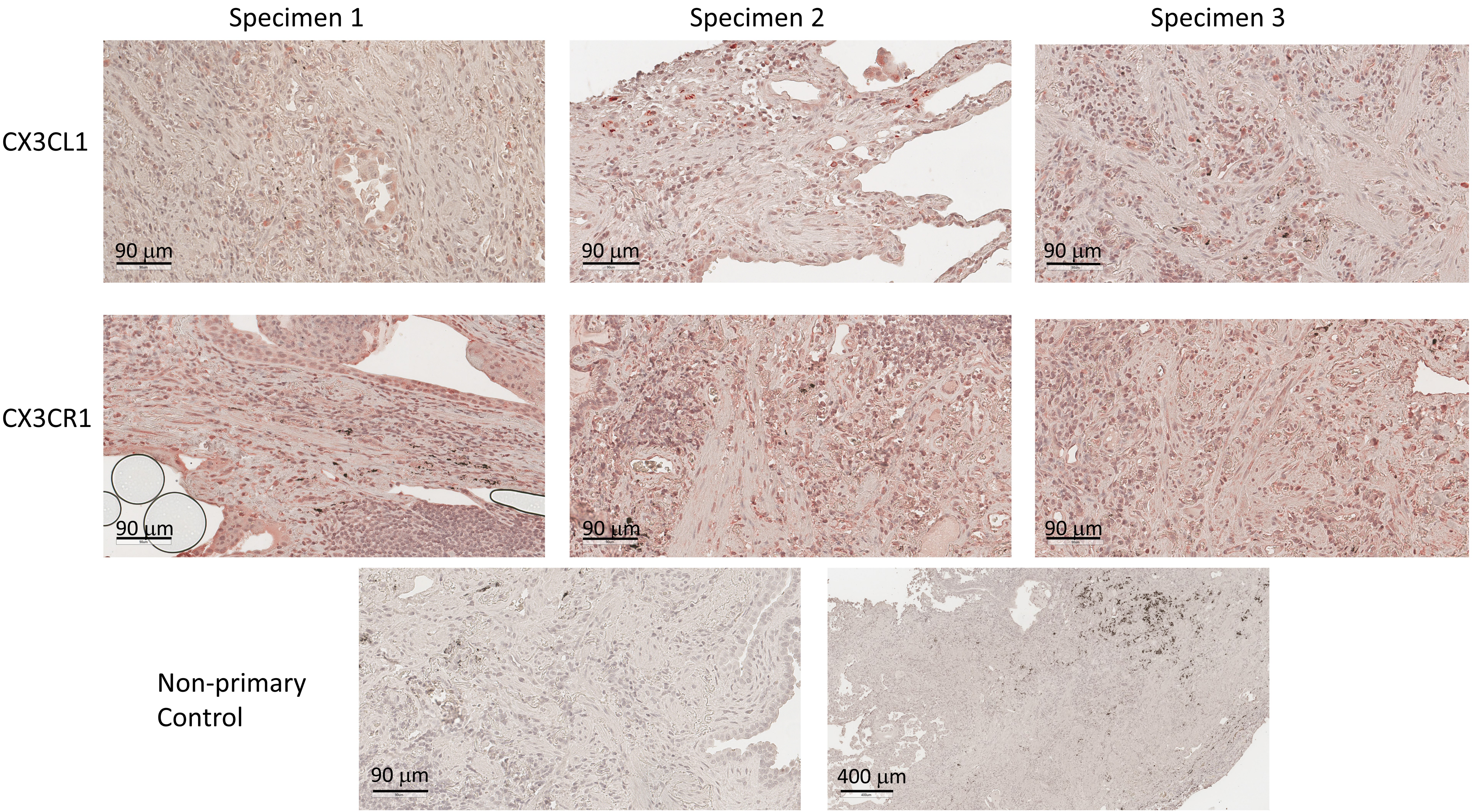

Supplement: Supplementary file 1 — Supplementary figure S1. [file ijmsv17p2357s1.pdf]
